# Molecular species delimitation refines the taxonomy of native and nonnative physinine snails in North America

**DOI:** 10.1038/s41598-021-01197-3

**Published:** 2021-11-05

**Authors:** Michael K. Young, Rebecca Smith, Kristine L. Pilgrim, Michael K. Schwartz

**Affiliations:** 1 USDA Forest Service, National Genomics Center for Wildlife and Fish Conservation, Rocky Mountain Research Station, 800 E. Beckwith Avenue, Missoula, MT 59802 USA; 2 Present Address: Department of Ecology & Evolutionary Biology, University of Tennessee, 569 Dabney Hall, Knoxville, TN 37996 USA

**Keywords:** Phylogenetics, Taxonomy, Biodiversity, Invasive species

## Abstract

Being able to associate an organism with a scientific name is fundamental to our understanding of its conservation status, ecology, and evolutionary history. Gastropods in the subfamily Physinae have been especially troublesome to identify because morphological variation can be unrelated to interspecific differences and there have been widespread introductions of an unknown number of species, which has led to a speculative taxonomy. To resolve uncertainty about species diversity in North America, we targeted an array of single-locus species delimitation methods at publically available specimens and new specimens collected from the Snake River basin, USA to generate species hypotheses, corroborated using nuclear analyses of the newly collected specimens. A total-evidence approach delineated 18 candidate species, revealing cryptic diversity within recognized taxa and a lack of support for other named taxa. Hypotheses regarding certain local endemics were confirmed, as were widespread introductions, including of an undescribed taxon likely belonging to a separate genus in southeastern Idaho for which the closest relatives are in southeast Asia. Overall, single-locus species delimitation was an effective first step toward understanding the diversity and distribution of species in Physinae and to guiding future investigation sampling and analyses of species hypotheses.

## Introduction

It has been argued^1^ that species constitute the only level of the classification of life—systematics and taxonomy—that has objective reality. Often, however, the most fundamental characteristic of an organism from a human perspective is its name^1^. When we delineate a species and give it a name, we facilitate communication about its relation to its environment and to other species (ecology), its patterns of survival and activity (demography and life history), and its evolutionary history and distribution (phylogenetics and phylogeography)^2^. We tend to focus conservation actions on named taxa^3^, with the tacit assumption that their members can be unambiguously tallied as present or absent, their abundance estimated and monitored, and their status as native or introduced known. Yet all species and their names are hypotheses subject to acceptance, revision, or rejection, and discerning when a name represents one species, a few populations within a species, or a complex of species is crucial to taxonomy and conservation.

Robustly defining species among gastropods has been a particular challenge. Many are small and the taxonomy has generally been based on characteristics we can see, i.e., shell or soft-part morphology, which have been shown to vary in response to environmental factors or exhibit extreme conservatism among evolutionary lineages, and thus be of uncertain value for diagnosing species^4–7^. Consequently, species hypotheses and their higher-order assignments to genera, families, and orders could generously be described as fluid, and even authorities attempting to categorize extant species have not reached consensus, e.g., contrast^8^ with^9^. Although geography can also be informative with regard to species delineation, the recent history of many gastropods involves their widespread translocation by humans^10,11^ to the extent that their continental origins are sometimes uncertain^12^ and what were thought to be rare local endemics are instead members of globally invasive species^13–15^. At the opposite extreme, many recognized taxa are legitimately regarded as endangered because they are known from only one or a handful of sites and are restricted to freshwater habitats that are typically extensively modified for human uses^16^. Consequently, establishing valid species hypotheses is a critical issue.

Increasingly, molecular tools are being applied to resolve taxonomic and conservation issues among taxonomic divisions within gastropods^17^, but this has also been unevenly applied and contentious. Although the mitochondrial genome, often the workhorse for phylogenetic efforts because of its successful application to the majority of animal taxa^18^, has been problematic for revealing deep phylogenetic structure in gastropods^19–21^, it has been effective for detecting relationships among genera^22^ and for assignment of individuals to species^10^, the latter the primary goal of DNA barcoding^23^. Mitochondrial sequences are increasingly used as a first approximation for delimiting species among taxonomically challenging groups^24^, but this application is rendered more difficult in analyses of gastropods because of uncertainty about what constitutes the transition from intraspecific variation to interspecific differences. For examples, some authors view combinations of highly divergent lineages (genetic distances > 15%) as constituting a single taxon^7,25^, whereas others^6,26,27^ favor thresholds for interspecific divergence akin to those applied to vertebrates (e.g., 1–4%)^18,28^.

Gastropod snails in the subfamily Physinae (Physidae) have long posed a problem for taxonomists. Although they constitute a clade within a strongly supported, monophyletic Physidae in mitochondrial trees (Supplemental Fig. 1)^9^, species- and genus-level membership in Physinae remain unstable. At one time, all members were placed in the genus *Physa*, but subsequently have been variously assigned to *Beringophysa*, *Haitia*, *Costatella*, *Petrophysa*, *Physella*, *Sibirenauta*, *Utahphysa*, and *Stenophysa*, the latter sometimes grouped with members of the genus *Aplexa* in the subfamily Aplexinae^8^ or left unassigned^29^. Unsurprisingly, there are also substantial differences among different authors with respect to the identity and number of physinine species in North America^9,30,31^, even among scientific bodies charged with maintaining a valid taxonomy (Supplemental Table 1). In part, this may have arisen because some physinine snails are ecological generalists whose appearance is plastic in response to environmental characteristics and the presence of predators^4,32^. All are capable of self-fertilization, which can contribute to rapid evolution and lead to the long branches associated with Physinae in several phylogenies^21^. Such divergence may be accentuated in isolated or thermally enhanced habitats where founder effects are pronounced, populations are small, and generation times may be short^33^.

Members of the physinine fauna of the Snake River basin in southern Idaho, USA are exemplars of many of these taxonomic issues. Taylor^34^ first described *Physa* (*Haitia*) *natricina* (hereafter, *Physella natricina*) as having a restricted distribution in a portion of the Snake River main stem, and shortly thereafter the species was listed under the US Endangered Species Act^35^. Authors of a subsequent morphological study of thousands of specimens from the Snake River^36^ argued that *P. natricina* did not constitute a valid taxon, and that all specimens from the Snake River were instead *P. acuta*, at the time thought to be introduced from Europe where it was first described in 1805. Only more recently was it recognized that the globally invasive *P. acuta* was actually indigenous to North America^12^. Regardless of its origins, its presence in the Snake River was questioned in a subsequent study of newly acquired and museum specimens from the Snake River^37^, whose authors countered that not only was *P. natricina* sufficiently morphologically and genetically discrete to merit recognition, but that the thousands of other specimens in their dataset were *P. gyrina*, not *P. acuta*. Adding to the regional complexity is a candidate species from a spring complex in Oregon in the Owyhee River basin, a tributary to the Snake River, that appears to constitute a valid taxon that has yet to be described^38^, and the suspected presence of an unknown number of introduced species as well as native species of dubious validity^30^.

Initially, we planned to use molecular tools to perform specimen assignment on a sample of unidentified physinine gastropods from the Snake River basin in Idaho, USA, that were collected as part of an application for hydropower relicensing (Michael Stephenson, Idaho Power Company, personal communication). The diversity of lineages we encountered, including the presence of several potentially new species, required a broader phylogenetic scope. Hence, our objectives were to conduct molecular species delimitation among Physinae from this region and in public databases using a single mitochondrial locus and variety of approaches, to corroborate those analyses for locally obtained samples with sequences of a single nuclear gene, and to assign specimens to species using molecular tools to understand the geographical characteristics of the evolutionary lineages.

## Results and discussion

**Figure 1:**
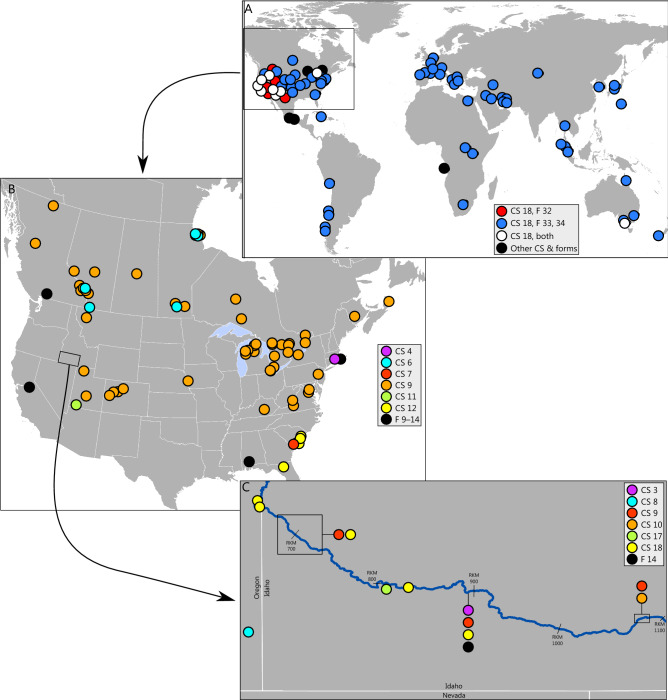
Distribution of candidate species (CS) and forms (F) of members of Physinae. (**A**) Members of the *Physella acuta* sensu lato complex (CS 13–18, F 22–26). (**B**) Members of all other candidate species and forms in the US and Canada, excluding specimens from the Snake River basin. (**C**) Members of candidate species and forms found in the Snake River basin, Idaho-Oregon. The base maps were initially prepared in ArcGIS (https://www.arcgis.com/index.html) and modified in Inkscape 1.1 (https://inkscape.org).

**Figure 2:**
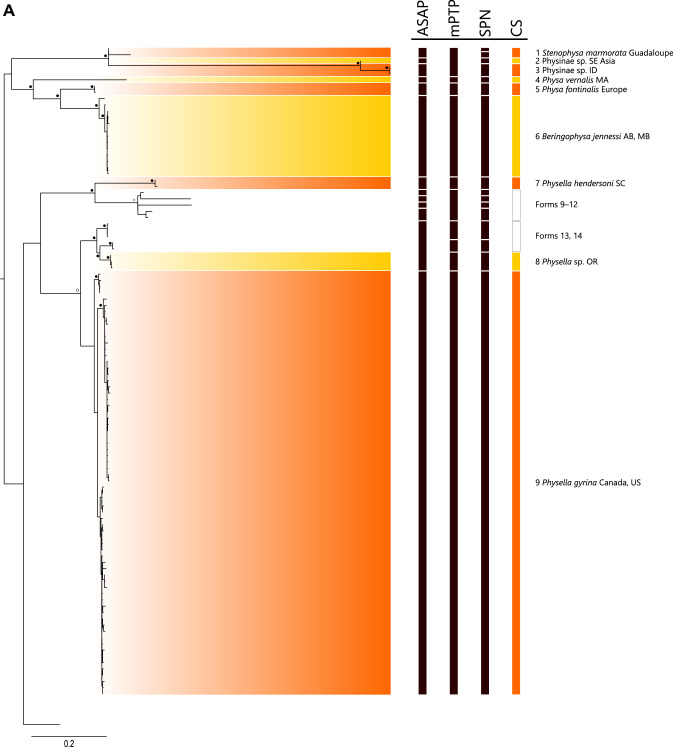

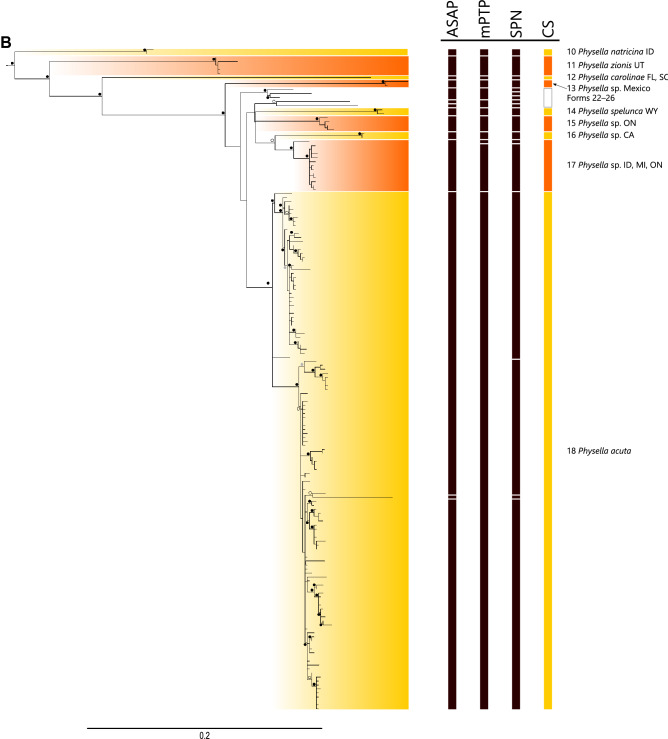
Maximum-likelihood phylogeny of Physinae based on COI haplotypes and the results of species delimitation analyses. CS denotes candidate species; species labels are in Table 1 and sequence labels are in Supplemental Fig. 2. Dots (white, 85–90%; gray, 90–95%; black, > 95%) denote ultrafast bootstrap support.

Species delimitation methods were relatively consistent and often corroborated the current taxonomy but recognized greater diversity (Fig. 2, Supplemental Fig. 2). The best-scoring ASAP analysis delimited nine species, but its distance threshold (14%) was more typical of intergeneric rather than interspecific distances and tended to combine well-established and divergent taxa, e.g., one candidate species consisted of specimens of *Physa fontinalis*, *Beringophysa jennessi*, *Physella pomilia*, and *Physella gyrina*. Three of the ten top-scoring models had distance thresholds of 5.17, 5.57, and 6.28% and delineated 25, 24, and 22 species; we chose the first as the most plausible initial estimate of species diversity (see Supplemental File 1). Statistical parsimony network analyses generated 34 independent networks at the 90% threshold. Higher threshold values generated higher numbers of independent networks (e.g., 95% threshold, 39 species; data not shown) that were less consistent with the other methods and the existing taxonomy. Analyses using multi-rate Poisson tree processes delineated 22 species, albeit sometimes in combinations of specimens unsupported by the other analyses. For example, one candidate species consisted of all members of the first major clade in Physinae, despite that the maximum intraspecific distance was 23.7%. The maximum-likelihood phylogeny of histone sequences (Supplemental Fig. 3) offered less resolution among candidate taxa, but still recovered Physinae as a monophyletic clade represented by four distinct groups, with two groups representing single candidate taxa (CS 3 and 8) and two representing multiple candidate taxa (one composed of CS 9 and an undelimited taxon, the other of CS 10 and 18). Taking into account all lines of genetic, morphological (based on field identifications), and geographical evidence (Fig. 1, Supplemental Figs. 2–4, Supplemental Table 2), we delimited 18 candidate species (Table 1). Specimen assignment to a candidate species was usually straightforward; only four of the additional 232 specimens that were considered could not be assigned. Inclusion of additional specimens caused shifts in the within-tree position of clades representing candidate species, but rarely of general levels of bootstrap support for them (Supplemental Fig. 5).

**Table 1:** Candidate species of Physinae delimited in this analysis; members are in Fig. 2, Supplemental Fig. 2.

Candidate species	Form	Taxon	Method			Diagnosed		Distance		BS
			ASAP	mPTP	SPN	AA	H3	Intra- specific	Inter- specific	
1	1–2	*Stenophysa marmorata*	x	—	—	x		4.8	15.0	—
2	3	Physinae sp. SE Asia	x	—	x	x		—	7.0	—
3	4	Physinae sp. ID	x	—	x	x	x	0.2	7.0	100
4	5	*Physa vernalis*	x	x	x	—		—	15.0	—
5	6	*Physa fontinalis*	x	x	x	—		0.2	9.4	100
6	7	*Beringophysa jennessi*	x	x	x	—		3.4	9.4	98
7	8	*Physella hendersoni*	x	x	x	—		0.5	12.8	100
—	9	*Physella* sp. AL	—	—	x	—		—	3.6	—
—	10	*Physella* sp. AL	x	—	x	—		—	8.9	—
—	11	*Physella* sp. VA	x	—	x	—		—	9.8	—
—	12	*Physella* sp. CT	—	—	x	—		2.0	3.6	77
—	13	*Physella* sp. CA	—	x	x	—		0.4	4.3	100
—	14	*Physella* sp. BC, ID	—	x	x	—	x	0.4	4.1	100
8*	15	*Physella* sp. OR	—	x	x	x	x	0.4	4.1	100
9	16–17	*Physella gyrina*	x	x	x	—	x	4.1	6.4	64
10	18	*Physella natricina*	x	x	x	—		0.5	12.7	100
11*	19	*Physella zionis*	x	x	x	—		1.8	12.7	100
12	20	*Physella carolinae*	x	x	x	x		—	14.3	—
13	21	*Physella* sp. Mexico	x	x	x	x		2.0	10.5	100
—	22	*Physella* sp. CA	—	—	x	—		—	2.7	—
—	23	*Physella* sp. ON	—	—	x	—		—	3.4	—
—	24	*Physella* sp. ON	—	—	x	—		—	2.7	—
—	25	*Physella* sp. Angola	x	—	x	—		—	6.8	—
—	26	*Physella* sp. Mexico	x	—	x	—		—	8.0	—
14*	27	*Physella spelunca*	x	x	x	x		0.9	8.6	100
15	28	*Physella* sp. ON	x	x	x	—		1.4	7.1	100
16	29	*Physella* sp. CA	x	x	x	x		0.2	7.1	100
17	30–31	*Physella* sp. ID, MI, ON	x	—	—	—		3.2	5.5	100
18	32–34	*Physella acuta*	—	x	—	—		9.4	5.2	100

A dash indicates that evidence was insufficient to delimit a taxon. Form refers to the statistical parsimony network group (Supplemental Table 2). Where the present taxonomy is consistent with a candidate species, its name is provided. An “x” denotes that a species or form was delimited by a method, a dash that it was not. Diagnosed (denoted by an “x”) indicates that a candidate species had a diagnostic COI AA sequence (relative to all other candidate sequences or forms) or H3 sequence (relative to *Physella acuta*, candidate species 18). Distances, based on the simple number of nucleotide differences, are the maximum intraspecific distance and the minimum interspecific distance; a dash indicates that a candidate species or form was represented by a single haplotype or specimen. Bootstrap support (BS) is provided for monophyletic clades; a dash indicates that the group was either not monophyletic or represented a singleton. Candidate species marked with an asterisk are local endemics only known from one location.

The geographical distribution of candidate species often did little to inform species boundaries (Fig. 1). There was widespread range overlap among candidate species, and more than one proposed taxon was often collected at a single location. Sometimes, candidate taxa were unlikely to be indigenous to the only location in which they were found (see below), implying that introductions have been widespread. This was further emphasized by the distribution of specimens in the Snake River (Fig. 1C), in which the highest diversity of candidate species was collected immediately downstream from the reach featuring a high concentration of aquaculture facilities.

Below, we review these candidate species in their order of appearance in the maximum-likelihood phylogeny used in species delimitation. Monophyletic groups insufficiently diverged to constitute candidate taxa in our analyses, but recognized by some methods, were considered forms and labeled with their statistical parsimony network designation (Table 1, Supplemental Fig. 2, Supplemental Table 2) for discussion. We also note one group excluded from species delimitation—because of insufficient sequence length—that appeared in the specimen assignment phylogeny.

### *Stenophysa* and unassigned taxa

This highly supported (BS 96) clade was sister to and highly divergent from nearly all other members of Physinae. It included two specimens of *Stenophysa marmorata* (CS 1) from the Caribbean that constituted a robustly delimited species according to most methods. Also in this clade, however, were two specimens from southeastern Asia and a handful of specimens from one site (river kilometer 899) on the Snake River. Although these specimens group with *Stenophysa marmorata*, this may be a consequence of long-branch attraction. In the COI amino acid phylogeny, the unidentified specimens constitute a cohesive clade that is sister to all other members of Physinae and does not group with *S. marmorata*. Ng et al.^14^ recognized the novelty of the southeastern Asian specimens, rejected that they were introduced forms of *S. spathidophallus*^8^, and opined that they represented a new species. We take this a step farther in suggesting that the specimens from southeastern Asia and those in the Snake River constitute sister but separate candidate taxa (CS 2 and 3, respectively; minimum interspecific distance, 7.0%). Ironically, it seems possible that the specimens in the Snake River that constitute a new species are introduced, given their position immediately downstream from aquaculture facilities and their divergence from all other specimens of Physinae in North America (minimum interspecific distance, 23.4%). Moreover, this level of divergence is more consistent with their assignment to a separate genus, but whether this should be *Stenophysa* or a new genus requires additional samples and more comprehensive genetic analyses. A single specimen from Japan (GenBank accession LC381493, identified as *Physella acuta*) also grouped with CS 3 in the histone phylogeny, but could not be assigned to that species because it lacked a COI sequence and because there are no comparable histone sequences of representatives of CS 1 and 2.

### *Physa* and *Beringophysa*

Another group of taxa forming a strongly supported (BS 96) clade consisted of one European and two North American members delimited as candidate species—*Physa vernalis*, *P. fontinalis*, and *Beringophysa jennessi* (CS 4–6). A fourth recognized species, *P. skinneri*, lacked sequences of sufficient length for species delimitation, but formed a weakly supported (BS 80) clade in the specimen assignment tree, assuming that a specimen of *B. jennessi* (GenBank accession KM612096) is a misidentification. Others^9^ have discouraged the use of *Beringophysa* because they did not regard its members as sufficiently divergent to warrant placement in a new genus. Our results with a larger dataset support that conclusion, and we designate all members of this clade as *Physa*.

### *Physella* clade 1: *Physella hendersoni* and *Physella pomilia*

A weakly supported (BS 79) but monophyletic lineage is sister to the two previously described lineages, and can be thought to represent members of the genus *Physella*^9^. Within this are three better supported clades, the first of which (BS 98) was restricted to the southeastern US and for which species delimitation was only partly successful. Within this clade, one candidate taxon—*P. hendersoni* (CS 7)—was readily recognized because it was delimited in every analysis. Although *P. hendersoni* and *P. pomilia* have sometimes been synonymized because they will introgressively hybridize in captivity^39^, reproductive isolation is only one line of evidence about species hypotheses^40^, and on other grounds—molecular divergence, diagnostic COI amino acid sequences for *P. pomilia*, and hypothesized differences in their geographic distribution^39^—the two taxa are distinct. Whether *P. pomilia* constitutes one species or a species complex, however, is uncertain. Specimens belonging to the clade containing *P. pomilia* were highly divergent and delineated as one to four candidate taxa (forms 9–12) by the different methods. If treated as a single species under that name, this group would exhibit levels of intraspecific variation (up to 14.1%) that are inconsistent with most gastropod species boundaries. We accept *P. pomilia* as a valid name for some members of this lineage, but pending further sampling from this region, we left this group of specimens unresolved.

### *Physella* clade 2: the *Physella gyrina* complex

This represented a modestly supported (BS 88) clade that consisted of specimens identified either as one of a host of formerly valid nominal species or as a previously recognized but still unnamed candidate taxon^38^, and were not identified by their collectors. This was further divided into two subclades, the first of which was highly supported (BS 98) and consisted of three forms (13–15) from western North America that were either grouped into a single species or regarded as three separate taxa in different analyses. Each group had little intraspecific variation (all 0.4%) and strong bootstrap support (100), and differed from a sister taxon by 4.1 to 4.3%. One of these (form 15) is known to be morphologically distinctive and restricted to a single spring complex in eastern Oregon (and was included in our sample of new specimens), informally named the Owyhee wet-rock physa^38^; it also exhibited an amino acid COI sequence divergent from all other members of Physinae and a diagnostic H3 sequence. On those grounds, we recognize it as a candidate taxon (CS 8). We did not designate the other two forms as candidate taxa because we lacked comprehensive information on the distribution of each. One form (14), not assigned to a nominal species by its collectors, had previously been observed in the Similkameen River basin in British Columbia, and we detected individuals with an identical sequence at one site (river kilometer 899) in the Snake River. The other form (13) was labelled as *P. gyrina* and has been observed in two tributaries in the San Joaquin River basin in California. Determining whether these constitute independently evolving lineages warranting recognition or are part of a single diverse and widely distributed species requires further sampling and analysis, but neither represents *P. gyrina* sensu stricto.

Another candidate species (CS 9) was delimited in nearly all analyses, albeit with weak bootstrap support (BS 64) in the species delimitation phylogeny but very strong support (BS 99) in the species assignment phylogeny. It was represented by specimens from across much of Canada and the northern US (Fig. 1B). This included members of many nominal species—*P. ancillaria*, *P. aurea*, *P. brevispira*, *P. globosa*, *P. gyrina*, *P. johnsoni*, *P. magnalacustris*, *P. microstoma*, *P. parkeri*, *P. wolfiana*, *P. wrighti*, and *Utahphysa microstriata*—from at or near their type locations. Because the first member of this lineage to be described was *P. gyrina* from the Boyer River basin in Iowa, we follow Wethington and Lydeard^9^ and consider this candidate species to represent *P. gyrina*. Specimens identified as *P. johnsoni* from a spring complex in Alberta also had a diagnostic COI amino acid sequence, but the nucleotide sequences suggested little divergence from *P. gyrina* overall (minimum pairwise difference, 0.5%). This is consistent with recent colonization of this location following continental deglaciation^41^.

### *Physella* clade 3: *P. natricina*, *P. zionis*, *P. carolinae*, and the *P. acuta* complex

Three strongly supported clades in both phylogenies were resolved by all methods as three candidate taxa—*P. natricina* (CS 10) from deep-water habitats in the main-stem Snake River in Idaho, *P. zionis* (CS 11) from seeps and springs along the North Fork Virgin River in Utah, and *P. carolinae* (CS 12) from South Carolina and Florida. All three candidate species are morphologically and geographically distinctive^37,42^, and do not appear closely related to one another (minimum pairwise distances, 12.7–14.3%), although *P. natricina* and *P. zionis* do share a diagnostic COI amino acid sequence.

All remaining specimens and sequences belong to a highly supported (BS 100) clade that can be construed as *P. acuta* sensu lato. It contains specimens from many portions of North America, where this species is thought to be indigenous, and from across the globe where it has been introduced^12^. Most specimens were identified as *P. acuta* by their collectors, but others were nominally regarded as species with geographically restricted distributions, were identified as *P. gyrina*, or were unidentified. Wethington and Lydeard^9^ proposed treating all specimens, save those representing *P. spelunca*, as one highly divergent species, *P. acuta*. The phylogenetic patterns that we observed, however, suggest that *P. acuta* may represent a species complex harboring cryptic taxa, many of which require further sampling to resolve whether they are members of *P. acuta* or constitute independent taxa. Extensive geographical overlap among these candidate taxa (Fig. 1), however, renders this a challenging task, and may favor the more conservative interpretation.

One clade within this group that met all criteria for species recognition was represented by three specimens from Tlaxcala state in Mexico. These differed by 10.5% from all other members of this species complex and were not represented by specimens in any other portion of North America or the world. These specimens were only identified to family by their collectors, but assigned with high support to this species complex. Despite the limited sampling, we regard these as members of a new candidate taxon (CS 13), in part because Taylor^8^ considered southern Mexico to have a number of indigenous taxa that have been poorly inventoried. Likewise, there was consensus among all methods for recognizing *P. spelunca*, a candidate species (CS 14) which differed by 8.6% from its nearest neighbor and is restricted to a single spring in the Bighorn River basin in Wyoming. Two other groups meeting the same standards were represented by specimens restricted to portions of Ontario (CS 15) and specimens from two locations in California (CS 16), both with minimum interspecific distances of 7.1%. An additional clade (CS 17) was highly supported (BS 100) but less geographically constrained; although most specimens were found across Ontario, single specimens were also collected in Michigan and Idaho. The nearest-neighbor distance was 5.5%, and despite being a highly supported member of the *P. acuta* species complex, all specimens were identified by their collectors as *P. gyrina*, implying that they were morphologically distinctive.

Finally, three additional clades were delimited as candidate species by some but not all methods, and their geographical locations or limited representation made interpretation difficult. One is represented by a single haplotype in Angola (form 25), which seems unlikely to represent its historical range, a second was represented by a single specimen from Mexico (form 26), and a third (forms 22–24) was represented by three haplotypes, two from Ontario (of which one, GenBank accession EU038360, is from the type location of *P. billingsii*^9^) and one from California. Recognizing these last three as valid taxa is not warranted without additional sampling to understand their distributions.

All remaining specimens constituted a strongly supported (BS 100) clade that most methods delimited as *P. acuta* (CS 18). These were divisible into two strongly supported clades (BS 99, 100) with distinctive ranges. Ebbs et al.^43^ also observed two clades, one of which was globally distributed, including throughout the US, whereas the other was restricted to the western US. We found the same geographic pattern (Fig. 1), although one specimen grouping with the western US clade (GenBank accession MG976099, submitted subsequent to^43^) was also present in Australia. Although treated as a single species here, the mitochondrial genomes of individuals representing each clade differed in length by 176 bases, and their mitogenomic nucleotide composition differed far more overall (9.92%)^43^ than did their COI sequences (4.18%), suggesting that our designation of species boundaries may be conservative. Nevertheless, none of the specimens representing other nominal species—*P. cubensis*, *P. cupreonitens*, *P. heterostropha*, *P. integra*, *P. niagarensis*, or *P. virgata*—exhibited species-level divergence or diagnostic amino acid sequences, and all specimens could be regarded as *P. acuta*^9^. This treatment, however, does combine individuals with substantial divergence (maximum intraspecific difference, 9.4%). Notably, the most divergent individual (AY651203) in both nucleotide (minimum interspecific difference, 5.5%) and amino acid sequences is from the Bighorn River in Wyoming in the vicinity of the type location of *P. spelunca*. This specimen was delimited as its own species in several analyses, but whether this constitutes an unrecognized and distinct lineage is unknown without further sampling.

## Conclusion

Our analyses indicate that species diversity within Physinae appears to be both underestimated and overestimated within some species complexes (Supplemental Tables 1, 2) because molecular species boundaries have been overlooked. The molecular delimitation approaches that we adopted are relatively conservative, thus our proposed taxonomy is likely to be robust albeit only a first step toward resolving the taxonomy of this group. More importantly, however, is that it is testable, and because these analyses are based on a single mitochondrial locus of samples from a limited spatial sample, these candidate species and forms represent a set of hypotheses warranting further evaluation. Regardless of that assessment, it is evident that this subfamily’s diversity in the Snake River is greater than expected. Counter to previous assessments^36,37^, the fauna includes both clades of the previously recognized *Physella acuta*, as well as *P. gyrina*, *P. natricina*, an undescribed species within the *P. gyrina* complex, and an unrecognized and likely nonnative species that may warrant placement in a separate genus. Whether any species other than *P. natricina* is native to the main-stem Snake River, however, is unknown. The extensive water development, recreational use, and aquaculture facilities associated with the Snake River and its reservoirs afford multiple pathways for cryptic invasions of non-native gastropods^10,44^. Systematic sampling of the entire basin and its habitats, and those in other basins throughout western North America, would be necessary to delineate the current distribution of the aforementioned lineages, and might shed light on the likely historical diversity.

Nevertheless, leveraging historical taxonomic work—and even achieving taxonomic stability—for some lineages of Physinae may be a daunting task. Morphological species delimitation of members of the Physinae, particularly those in the *P. gyrina* and *P. acuta* complexes, has been plagued by uncertainty and was often founded on conchological characteristics that are known to co-vary with habitat conditions or predator presence^4,32^. Consequently, assigning type specimens from historical collections to modern molecularly delineated species may not be possible, which renders many of the available names unusable. Obtaining new specimens from type locations may help, but it is also likely that the widespread distribution of members of this subfamily via anthropogenic introductions, with subsequent dispersal fostered by downstream drift, localized flooding, and bird- or mammal-assisted translocation^45,46^, may undermine efforts to delineate and recognize the historical fauna. Should these taxa be capable of introgressive hybridization, the phylogenetic patterns produced by the hybrid lineages might render impossible the delimitation of historical species diversity by any method, or even lead to the formation of new hybrid taxa.

The topological instability of the species delimitation and specimen identification phylogenies, particularly the order of appearance of specimens and modest variation in bootstrap support for particular candidate species, are reminders that despite broad geographic sampling among the Physinae, there remain taxa that have been undersampled or overlooked, and that our results are but a first approximation of species diversity. Despite that members of the *P. acuta* clade are among the most broadly sampled specimens of gastropods in the world, many members of this subfamily, including those in the *P. gyrina* complex in western North America, *Stenophysa* at its type location and areas where it has been presumably introduced^8,47^, the *P. pomilia* complex, and those nominally attributed to *P. acuta* itself in Mexico, Ontario, and California, require further sampling in order to clarify their evolutionary relationships. Genomic analyses, or at least sequencing of additional nuclear and mitochondrial gene regions, would lead to more robust conclusions with respect to the presence of cryptic taxa and potential species boundaries. Regardless, our results demonstrate that single-locus species delimitation is an effective first step for discovering unexpected diversity and reconciling different interpretations of gastropod species hypotheses^6,27^ (Supplemental Table 1). The results also emphasize, given the repeated misidentifications of specimens of Physinae collected from throughout the world and the recognition of spurious taxa that are instead introduced species or environmentally induced morphological variants, that genetic corroboration is mandatory for distributional and taxonomic studies of this group.

## Methods

### Study area

Geologically, the Snake River basin consists of two structural elements. The western half is a graben associated with basin and range faulting from the ongoing disassembly of the North American plate from its encounter with the Pacific plate, whereas the eastern half has resulted from subsidence following passage of the North American plate over the mantle plume associated with the Yellowstone hotspot^48^. Although the drainage pattern has been set since the capture of the Snake River by the Columbia River 2.5–3.0 million years ago^49^, the main-stem corridor underwent one of the largest freshwater floods every recorded 17.4 thousand years ago when Pluvial Lake Bonneville overtopped a watershed divide in southeastern Idaho and catastrophically drained in a flood that may have lasted for up to a year^50^. The resultant scour constructed the modern Snake River channel and would likely have extirpated any gastropods there. Lesser floods in tributary basins resulting from the failure of landslide dams over the last 100,000 years (e.g., in the Owyhee River^51^) would have had a similar effect, suggesting that the modern gastropod fauna of much of the Snake River basin arrived relatively recently from habitats unexposed to floodwaters or recent volcanism^52^. Nevertheless, the main-stem river and its tributaries host a relatively diverse endemic molluscan fauna and many of these taxa are considered at-risk^35^.

The gastropod fauna has also been shaped by more recent events. To support intensive municipal and agricultural development, many portions of the main-stem Snake River were dammed for flood control and power production or diverted for irrigation, which inundated habitats of native species and provided an array of novel habitats and corridors for invasive species^16^. Perhaps of equal relevance to gastropod diversity is that a portion of the basin (primarily from river kilometers 909 to 982; https://www.uidaho.edu/extension/county/twin-falls/aquaculture) has, for over a century, supported an extensive aquaculture industry focused on the production of food and ornamental fish. Elsewhere, aquaculture facilities and the aquarium trade have been the conduits for non-native gastropod species introductions world-wide^10,13,44^. Another concern is transport of non-native mollusks on recreational boats^53^, but the extent of the translocation of gastropods via this mechanism is unknown.

### Field samples and genetic analyses

From 2016 to 2019, biologists collected physinine snails (*n* = 190, preserved in 95% ethanol) from stream margin and deep-water habitats in and along a 436-km reach (river kilometers 642–1078) of the main-stem Snake River in southern Idaho and eastern Oregon (Fig. 1). These specimens were supplemented with six previously collected samples of *P. natricina*, two specimens of the unidentified candidate species from the Owyhee River basin^38^, and two specimens of *Fisherola nuttallii* (Lymnaeidae), which we planned to use as an outgroup, an approach we abandoned because of uncertainties about its relation to other Lancinae (Supplemental Fig. 1).

We used the QIAGEN DNeasy Blood and Tissue kit to extract genomic DNA from ground remains of each specimen, following the manufacturer’s instructions for tissue. We amplified a 622-base portion of the cytochrome c oxidase subunit 1 (COI) region of mitochondrial DNA (mtDNA) with a combination of published^54,55^ and custom-designed primers (PhysCOIF, ACAGGTTTAAGCTTRYTAATTCG; PhysCOIR, TGTAATAGCTCCAGCYAAAAC). Reaction volumes of 50 µL contained 50–100 ng DNA, 1× reaction buffer (Applied Biosystems), 2.5 mM MgCl_2_, 200 mM each dNTP, 1 mM each primer, and 1 U Taq polymerase (Applied Biosystems). The PCR program was 94 °C/5 min, [94 °C/1 min, 55 °C/1 min, 72 °C/1 min 30 s] × 34 cycles, 72 °C/5 min. We also amplified a 309-nucleotide segment of the nuclear H3 (histone) gene for representatives (*n* = 25) of each COI haplotype^56^. The quality and quantity of template DNA were determined by 1.6% agarose gel electrophoresis. The PCR product was cleaned using ExoSAP-IT™ PCR Product Cleanup Reagent (Life Technologies) and sequence data was generated at Eurofins Genomics (Louisville, KY) on an ABI3730XL sequencing machine. All nuclear and mitochondrial sequences were aligned in MAFFT 7^57^ with the default settings, manually adjusted as necessary, and converted to amino acids to obtain the correct reading frame and to ensure that stop codons were absent.

In addition to the above sequences, we downloaded all COI sequences (> 500 bases; *n* = 895) of members of Physinae from GenBank (https://www.ncbi.nlm.nih.gov/genbank/) and BOLD (http://www.boldsystems.org/index.php/databases; Supplemental Table 2). We also obtained the geocoordinates of each sequence from these public databases or from the published literature, or estimated geocoordinates where only narrative descriptions were provided. Most but not all US and Canadian species in this subfamily were represented in these collections. Specimens that were outliers in preliminary phylogenetic analyses were re-examined using the NCBI BLAST website (https://blast.ncbi.nlm.nih.gov); those most closely aligning with specimens other than Physinae were excluded from further analyses. We also downloaded all sequences (*n* = 156) of H3 of members of Hygrophila (Supplemental Table 3).

### Phylogenetic analyses, species delimitation, and specimen identification

We based species hypotheses on the phylogenetic species concept, for which reciprocal monophyly is the primary criterion^58^. We refined this to emphasize interspecific rather than intraspecific variation, for which we used the existing gastropod taxonomy where corroborated by molecular data as a guideline^59^. Although any fixed interspecific difference will fail for delineating some taxa^60^, minimum differences exceeding 5% are often characteristic of interspecific distances among many gastropod congeners (Supplemental File 1) and we used this value in our analyses. As our null hypothesis of species diversity and identity, we followed the naming conventions and taxonomy of^61^ and MolluscaBase (http://www.molluscabase.org, accessed 15 May 2021), which assigned most North American members of Physinae to *Physella* and synonymized many taxa with more broadly distributed forms. Where available, we evaluated specimens nominally assigned to formerly valid taxa that had been collected from at or near their type locations^9,31,62^ (Supplemental Table 2) for use as alternative hypotheses about species identities.

We used separate COI datasets for species delimitation and specimen identification. The first (*n* = 861 sequences, 561 bases) represented a compromise between maximal species coverage and inclusion only of sequences lacking missing data or ambiguous bases. This dataset was reduced to representative haplotypes (*n* = 270) using the online version of CD-HIT^63^. The second dataset consisted of all sequences of Physinae longer than 500 bases (*n* = 1093).

Our first step in species delimitation was to build a maximum-likelihood phylogeny to serve as the basis for subsequent analyses. We used the single-haplotypes version of the first dataset. For tree construction, we used IQ-TREE^64^ implemented via the CIPRES gateway (https://www.phylo.org/). We assigned three preliminary partitions based on codon position, then selected edge-linked partitions and the TESTMERGE setting to determine the best-fitting substitution models, which were TN + F + G4 (position one), F81 + F + G4 (position two), and TIM + F + G4 (position three). We assigned support values to the consensus maximum-likelihood tree based on 1000 ultrafast bootstraps. We used *Sibirenauta elongata* (GenBank accession HQ969868) as an outgroup.

We explored multiple lines of evidence to identify potential sets of candidate species based on the COI sequences. First, we used the online version (https://bioinfo.mnhn.fr/abi/public/asap/#) of ASAP (Assemble Species by Automatic Partitioning)^65^, a method similar to ABGD^66^ in which genetic distances are used to identify the transition between intraspecific variation and interspecific divergence, but which includes a scoring system to identify the best-fitting set of partitions, i.e., candidate species. We adopted the default values and distances based on the K80 substitution model because of its similarity to the traditional distance metric used in barcode-based analyses^24^. We did not select the best-scoring overall model (for which differences among candidate taxa were akin to intergeneric differences in other gastropod families^5,67^, but instead chose the best-scoring model for which the difference threshold was more consistent with congeneric, interspecific differences in the existing gastropod taxonomy^59^ (Supplemental Table 2). Second, we used the online version (https://mptp.h-its.org/#/tree) of maximum-likelihood multi-rate Poisson tree processes (mPTP)^68^, which used the phylogenetic tree from the maximum-likelihood analysis to identify the transition between species- and population-level divergence and can be less conservative than the other methods. Third, we analyzed sequences in TCS 1.21^69^ to construct 90% maximum parsimony networks. Although independent networks tend to constitute a conservative estimate of the number of candidate species of several animal taxa when using the 95% threshold^70^, the more relaxed threshold was in keeping with the greater intraspecific divergence associated with gastropods. Fourth, we re-visited the maximum-likelihood phylogeny, and considered strong support for candidate species as a bootstrap value > 85^71^ for a reciprocally monophyletic clade identified by one of the aforementioned approaches. Fifth, we used MEGA 7.0^72^ to build a pairwise distance matrix based on the absolute number of differences between sequences, then examined the maximum genetic distance among members of a candidate species and the minimum distance to a non-member^73^. If the latter exceeded the former (i.e., created a barcode gap)^24^ and the latter exceeded 5%, we considered this strong support for a candidate species. The sixth step was to build a maximum-likelihood tree in IQ-TREE of the COI sequences translated to amino acids (best-fitting model, mtMet + R3). Because amino acid variation among most sequences was low, bootstrap support for most clades was weak, and we only used this approach to examine whether sequences were diagnostic for clades detected in the other analyses, and only for those represented by the newly collected specimens. The final step was to propose a set of species hypotheses. When most methods delimited a clade (because consensus among all methods was unlikely)^74^, we designated it as a candidate species (Supplemental Fig. 6). Candidate species represented by a singleton haplotypes were not treated as distinct unless multiple specimens exhibited that haplotype or other specimens in the specimen-identification step grouped (< 2% difference, based on the simple number of differences) with that candidate species. We also considered whether a candidate species was geographically cohesive, because geography is often the most diagnostic characteristic of a species^75^. When spatial sampling was insufficient to determine whether forms represented distinct lineages or were part of a more widely distributed and diverse clade, we deferred from promoting forms to candidate species.

More sophisticated multi-locus species delimitation methods were not used, largely because the nuclear gene that we sequenced, H3, is uninformative for recognizing deep phylogenetic branch structure because it lacks parsimony-informative amino acid variation^76,77^, and because there were only two sequences of Physidae in public databases to use as references. Sequences of this gene can, however, diagnose species-level or higher groups^76^, therefore we built a maximum-likelihood phylogeny of Lymnaeoidea (with four members of Caenogastropoda as outgroups; Supplemental Table 3) in IQ-TREE with the aforementioned settings and the best-fitting evolutionary models for each codon position (positions one and two, TIM2e + I; position three, TVMe + I) to evaluate the placement and variation of specimens of Physinae and *Fisherola* in our sample and to corroborate species delimited in the mitochondrial analyses.

For specimen identification, we built a COI maximum-likelihood tree (with the aforementioned specifications) using the dataset containing all Physinae sequences. Specimens were assigned to a candidate species if they were members of the same monophyletic clade as a specimen or specimens with more complete data. Specimens that were members of clades not part of the original analyses—which included one formally recognized species—were not delimited in this analysis but were identified.

## Supplementary Information

Supplementary Information.

Supplementary Legends.

Supplementary Figure S1.

Supplementary Figure S2.

Supplementary Figure S3.

Supplementary Figure S4.

Supplementary Figure S5.

Supplementary Figure S6.

Supplementary Table S1.

Supplementary Table S2.

Supplementary Table S3.

## Data Availability

All newly generated sequences have been deposited in GenBank (COI, OK510580–OK510777, OK637077, OK637078; H3, OK559403–OK559425). All other data generated or analyzed during this study are included in this article and its supplementary information.
