# Supplementary material for: Molecular species delimitation refines the taxonomy of native and nonnative physinine snails in North America
Source: Sci Rep. 2021 Nov 5;11:21739. doi: 10.1038/s41598-021-01197-3 (PMC8571305; doi:10.1038/s41598-021-01197-3)

Supplemental File 1. Interspecific differences among gastropods: re-analyses of two datasets.

We re-analyzed datasets for two genera of gastropods for which molecular species delimitation was recently used to evaluate the accepted taxonomy. The first of these was the COI dataset of Strong & Whelan (2019) of all sequences (*n* = 639 with no outgroup; 658 bases) of *Juga* (Semisulcospiridae; Caenogastropoda) either submitted as part of their study or downloaded from GenBank. We further modified this dataset by removing all sequences with missing or ambiguous data (*n* = 12), and reduced it to representative haplotypes (*n* = 151) using the online version of CD-HIT (Huang et al. 2010). These data were then analyzed using the online version of ASAP (https://bioinfo.mnhn.fr/abi/public/asap/#, accessed 21 May 2021; Puillandre et al. 2020) with the K80 evolutionary model and the default settings. This is the same algorithm used by ABGD (Puillandre et al. 2012), but one that includes a scoring system to identify the most likely number of candidate species.

The original analysis using ABGD retrieved 24 species as the best-supported solution when using that evolutionary model and led the authors to reject its applicability for species delimitation (Strong & Whelan 2019, p. 100, column 2, second full paragraph). Using a host of additional analyses, other genes, and geographical and morphological information, Strong & Whelan (2019) instead concluded that *Juga* consisted of nine molecularly identifiable species. Likewise, our re-analysis of their data using ASAP returned 7, 8, or 10 as the most likely number of candidate species, with threshold interspecific differences of 3.82 to 5.25% (Figure 1). Although we demonstrated that the choice of an evolutionary model appeared to have little influence on these results, more importantly it demonstrated that COI sequences coupled with simple models of species delimitation can provide a robust initial estimate of species boundaries and diversity (Ratnasingham & Hebert 2013).

The second dataset consisted of COI sequences of *Galba* (Lymnaeidae; Heterobranchia), many of which were in a global dataset analyzed by Alda et al. (2021). They did not include all species in this genus, but did select from a broadly distributed sample and downloaded all available sequences from GenBank. They performed species delimitation using a multi-step process, multiple methods, and sequences of up to four genes including COI, and concluded that their dataset represented 6–9 species.

We repeated aspects of their analysis by downloading all available sequences of COI for members of *Galba*, *Fossaria*, and *Bakerlymnaea*; the latter two have been synonymized with *Galba* (Johnson et al. 2013). We then built a neighbor-joining tree to ensure that all sequences were likely members of *Galba*. Five outliers were re-assessed using the BLAST algorithm, and were found to be more closely related (percentage identity > 98%) to other gastropod genera (*Austropeplea*, *Hinkleyia*, *Ladislavella*, and *Mediappendix*) and were removed from further analyses. We also removed all sequences with missing or ambiguous positions, which excluded at least one species (attributed to *G. bulimoides*). The resulting dataset consisted of 251 sequences (628 bases), which was further reduced to 144 haplotypes of nine nominal species and one unidentified specimen using the online version of CD-HIT (Huang et al. 2010). Two of these species (*G. mweruensis* and *G. parva*) were not broadly included in Alda et al. (2021), nor was a distinct clade within *G. truncatula* from Japan (GenBank accessions LC360891–949). Using ASAP and the K80 evolutionary model with the default settings (Puillandre et al. 2020), the best-scoring models suggested the presence of 10–15 species with threshold distances among taxa of 3.00 to 5.55% (Figure 2). The reanalysis with three potentially new taxa is in line with the previous estimate of species diversity obtained by Alda et al. (2021).

Overall, the COI-based interspecific differences in *Juga* and *Galba* were similar, and comparable to those for other genera of gastropods that have been subject to molecular evaluation (Delicado et al. 2019; Liu et al. 2019). Hence, our adoption of 5% as a threshold for interspecific differences among taxa when using COI sequences provided a conservative, rational, and repeatable initial definition of species boundaries and one that allowed the use of the current taxonomy as a benchmark for recognizing species (Galtier 2019) among unstudied, cryptic, or poorly resolved groups.

**References**

Alda P, Lounnas M, Vázquez AA, Ayaqui R, Calvopiña M, Celi-Erazo M, Dillon Jr RT, Ramírez LC, Loker ES, Muzzio-Aroca J, Nárvaez AO. 2021. Systematics and geographical distribution of *Galba* species, a group of cryptic and worldwide freshwater snails. Molecular Phylogenetics and Evolution, 157, 107035.

Delicado D, Arconada B, Aguado A, Ramos MA. 2019. Multilocus phylogeny, species delimitation and biogeography of Iberian valvatiform springsnails (Caenogastropoda: Hydrobiidae), with the description of a new genus. Zoological Journal of the Linnean Society, 186, 892–914.

Galtier N. 2019. Delineating species in the speciation continuum: a proposal. Evolutionary Applications, 12, 657–663.

Huang Y, Niu B, Gao Y, Fu L, Li W. 2010. CD-HIT Suite: a web server for clustering and comparing biological sequences. Bioinformatics, 26, 680–682.

Johnson PD, Bogan AE, Brown KM, Burkhead NM, Cordeiro JR, Garner JT, Hartfield PD, Lepitzki DA, Mackie GL, Pip E, Tarpley TA. 2013. Conservation status of freshwater gastropods of Canada and the United States. Fisheries, 38, 247–282.

Liu HP, Hershler R. 2019. A new species and range extensions for three other species of pebblesnails (Lithoglyphidae, *Fluminicola*) from the upper Klamath basin, California–Oregon. ZooKeys, 812, 47–67.

Puillandre N, Brouillet S, Achaz G. 2020. ASAP: assemble species by automatic partitioning. Molecular Ecology Resources, 21, 609–620.

Puillandre N, Lambert A, Brouillet S, Achaz G. 2012. ABGD, Automatic Barcode Gap Discovery for primary species delimitation. Molecular Ecology, 21, 1864–1877.

Ratnasingham S, Hebert PD. 2013. A DNA-based registry for all animal species: the Barcode Index Number (BIN) system. PLoS ONE, 8, e66213.

Strong EE, Whelan NV. 2019. Assessing the diversity of western North American *Juga* (Semisulcospiridae, Gastropoda). Molecular Phylogenetics and Evolution, 136, 87–103.

Figure 1. Screen capture depicting ASAP results of re-analyses of sequences of *Juga*.


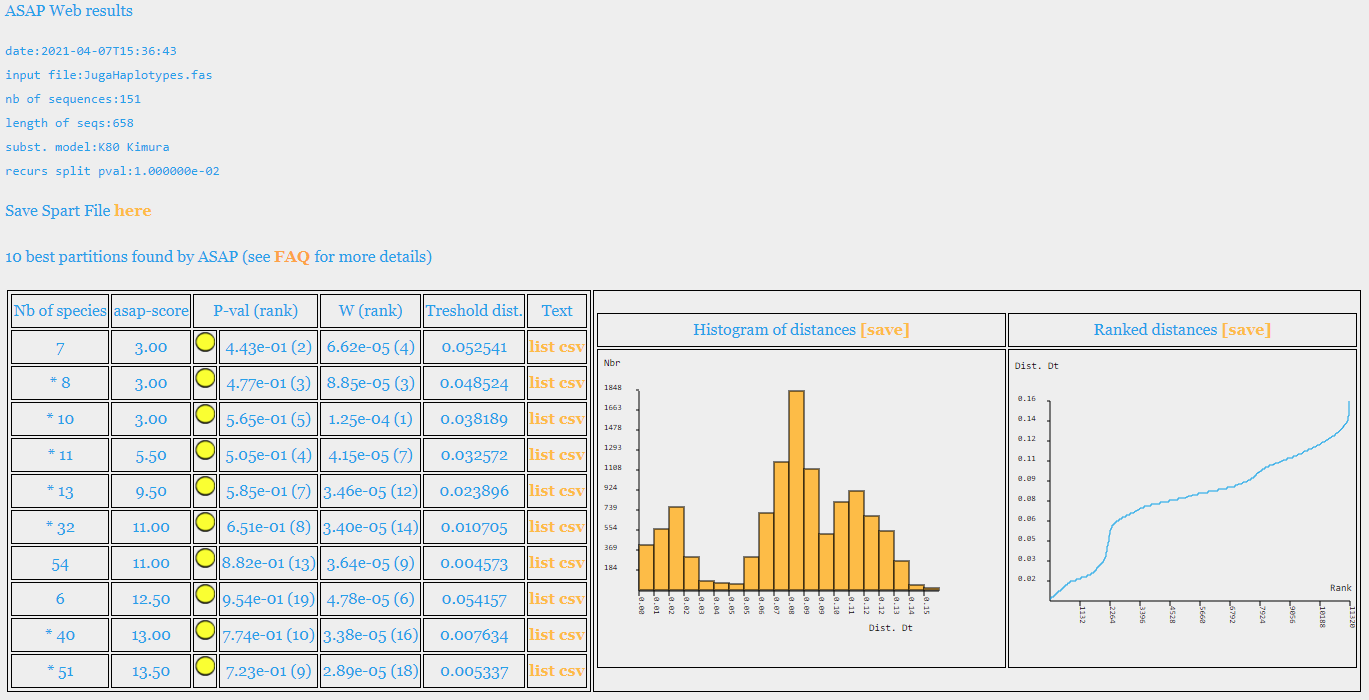


Figure 2. Screen capture depicting ASAP results of analyses of sequences of *Galba*.


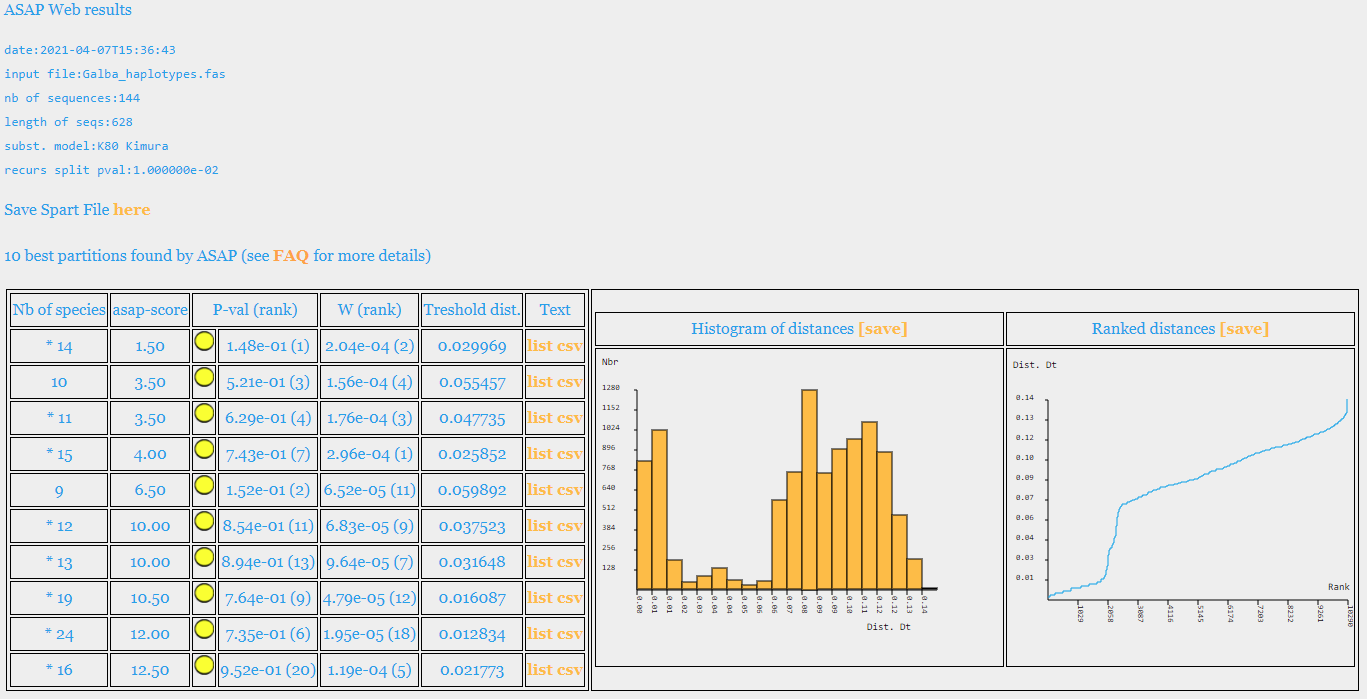

Supplement: Supplementary file 1 — Supplementary Information. [file 41598_2021_1197_MOESM1_ESM.docx]
