# Supplementary material for: Molecular species delimitation refines the taxonomy of native and nonnative physinine snails in North America
Source: Sci Rep. 2021 Nov 5;11:21739. doi: 10.1038/s41598-021-01197-3 (PMC8571305; doi:10.1038/s41598-021-01197-3)
Supplement: Supplementary file 2 — Supplementary Legends. [file 41598_2021_1197_MOESM2_ESM.docx]

Supplemental Figures.

Supplemental Figure 1. A maximum-likelihood phylogeny of Lymnaeoidea (Hygrophila; Gastropoda) based on COI sequences (*n* = 1,842 sequences of 622 bases) either of new specimens from this project or downloaded from public databases. For tree construction, we used IQ-TREE (Nguyen et al. 2015) implemented via the CIPRES gateway (https://www.phylo.org/). We assigned three preliminary partitions based on codon position, then selected edge-linked partitions and the TESTMERGE setting to determine the best-fitting substitution models, which were GTR+F+ASC+G4 (position one), TIM2+F+I+G4 (position two), and TPM3+F+I+G4 (position three), not adjusted for the amino acid reading frame. Ultrafast bootstrap support values ≥ 85 are shown. We used *Chilina mendozana* (GenBank accession KC347575) as an outgroup.

In this phylogeny, Physidae is a well-supported clade (bootstrap support (BS) 92) composed of two subclades representing the subfamilies Aplexinae (BS 99) and Physinae (BS 100), of which *Stenophysa* is part of the latter. Also note that *Fisherola nuttallii* (collected for this project and also represented by GenBank accessions HM230359, 60) and *Idaholanx fresti* (HM230356, 7; Campbell et al. 2017) are sister taxa that do not form a monophyletic clade with *Lanx alta* (HM230361, 2) or *Lanx patelloides* (HM230363) despite conchological similarities. These taxa do not constitute a monophyletic Lancinae (Lymnaeidae) in this analysis.

Supplemental Figure 2. Specimen identification based on a maximum-likelihood phylogeny of Physinae derived from COI sequences (*n* = 861; 561 bases) and the results of species delimitation analyses, with sequence labels provided. CS/form denotes candidate species or forms; species labels are in Table 1. Ultrafast bootstrap support values ≥ 85 are shown.

Supplemental Figure 3. Maximum-likelihood phylogeny of Hygrophila based on histone (H3) sequences, including all sequences in this study and from public databases (*n* = 179; 309 bases). Ultrafast bootstrap support values ≥ 85 are shown. We used four sequences of Caenogastropoda (GenBank accessions EU015835, FJ710383, MN322544, MN997736) as outgroups.

Supplemental Figure 4. Maximum-likelihood amino acid phylogeny of Physinae based on COI sequences (*n* = 861; 561 bases). For tree construction, we used IQ-TREE (Nguyen et al. 2015) implemented via the CIPRES gateway (https://www.phylo.org/). The best-fitting evolutionary model was mtMet+R3. Ultrafast bootstrap support values ≥ 85 are shown. We used *Sibirenauta elongata* (GenBank accession HQ969868) as an outgroup.

Supplemental Figure 5. Maximum-likelihood phylogeny of Physinae based on COI sequences, including all sequences in this study and from public databases (*n* = 1,093) with ≥ 500 bases. Ultrafast bootstrap support values ≥ 85 are shown.

Supplemental Figure 6. Flowchart illustrating how forms and candidate species were delineated. Two clades were exceptions to this logic. Candidate species 8 lacked a nearest-neighbor distance > 5%, but was well sampled, appeared to be restricted to single location, was morphologically distinct, and possessed a diagnostic COI amino acid sequence, thus was considered a candidate species from a total-evidence perspective. Form 25 met most criteria for designation as a candidate species, but was known to be introduced, lacked haplotype variation, and was part of the problematic *Physella acuta* complex, thus was considered to be inadequately spatially sampled. More thorough and systematic sampling of forms and candidate species is expected to refine these designations and reveal additional lineages.
