# Supplementary figures and images for: Molecular species delimitation refines the taxonomy of native and nonnative physinine snails in North America
Source: Sci Rep. 2021 Nov 5;11:21739. doi: 10.1038/s41598-021-01197-3 (PMC8571305; doi:10.1038/s41598-021-01197-3)

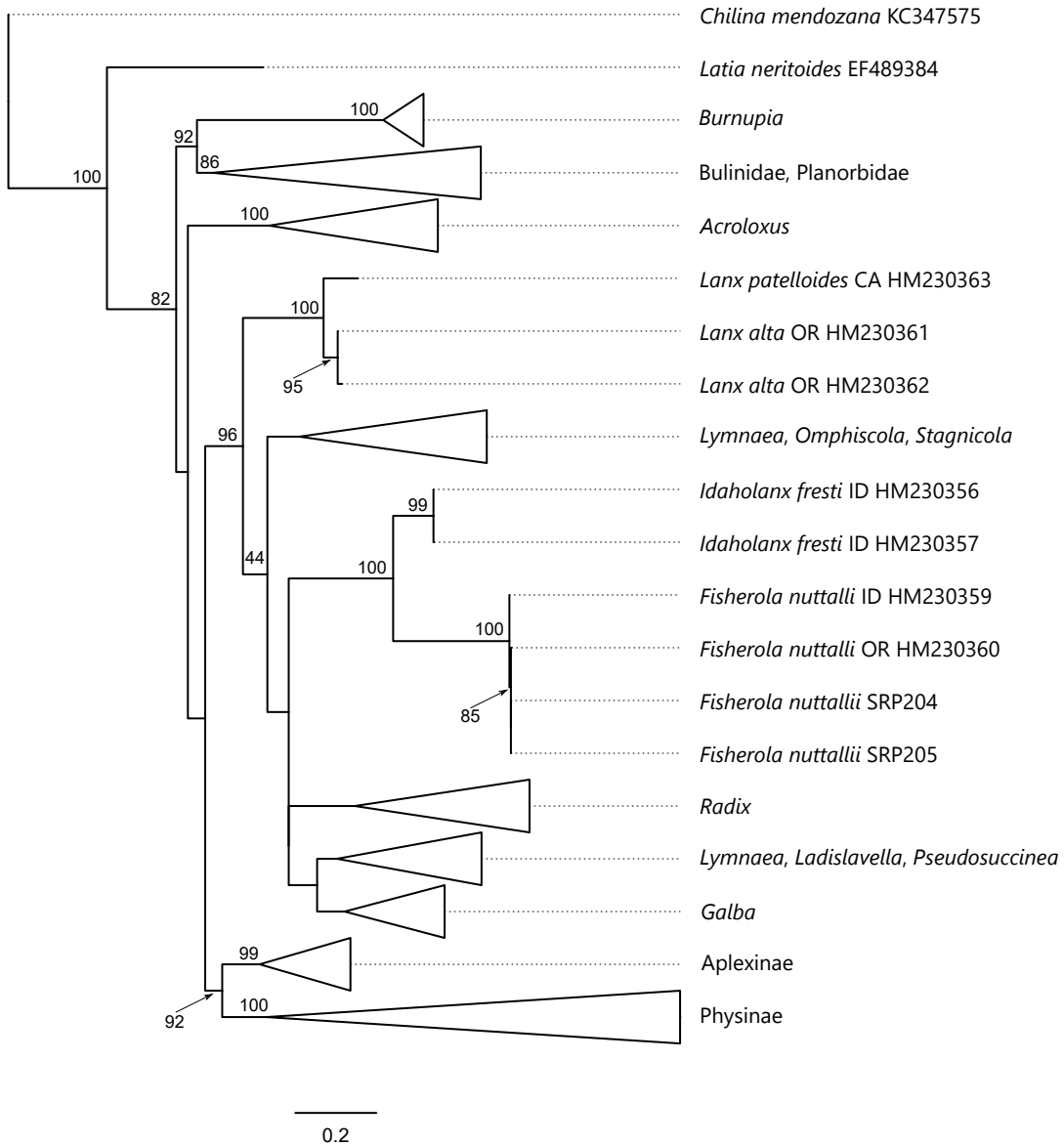

Supplement: Supplementary file 3 — Supplementary Figure S1. [file 41598_2021_1197_MOESM3_ESM.pdf]

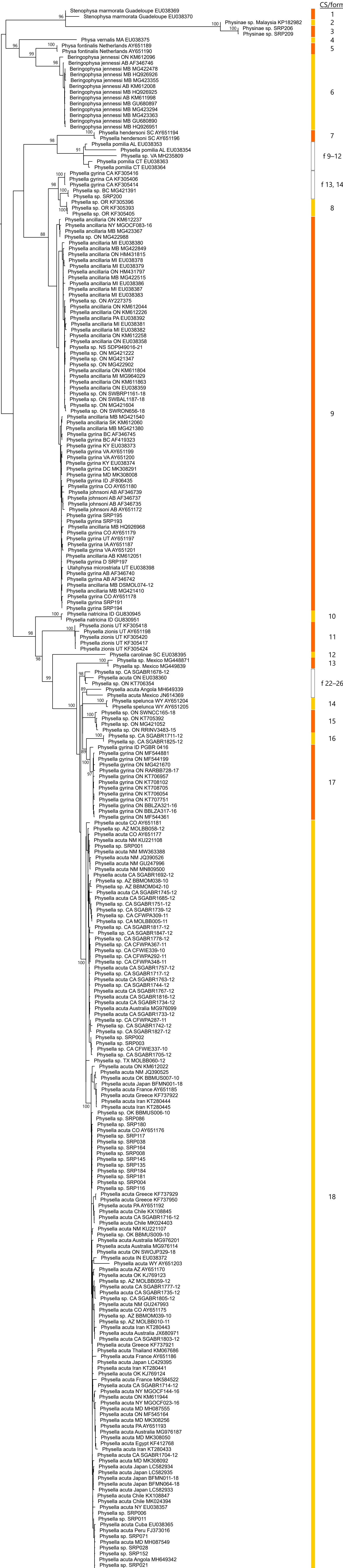

Supplement: Supplementary file 4 — Supplementary Figure S2. [file 41598_2021_1197_MOESM4_ESM.pdf]

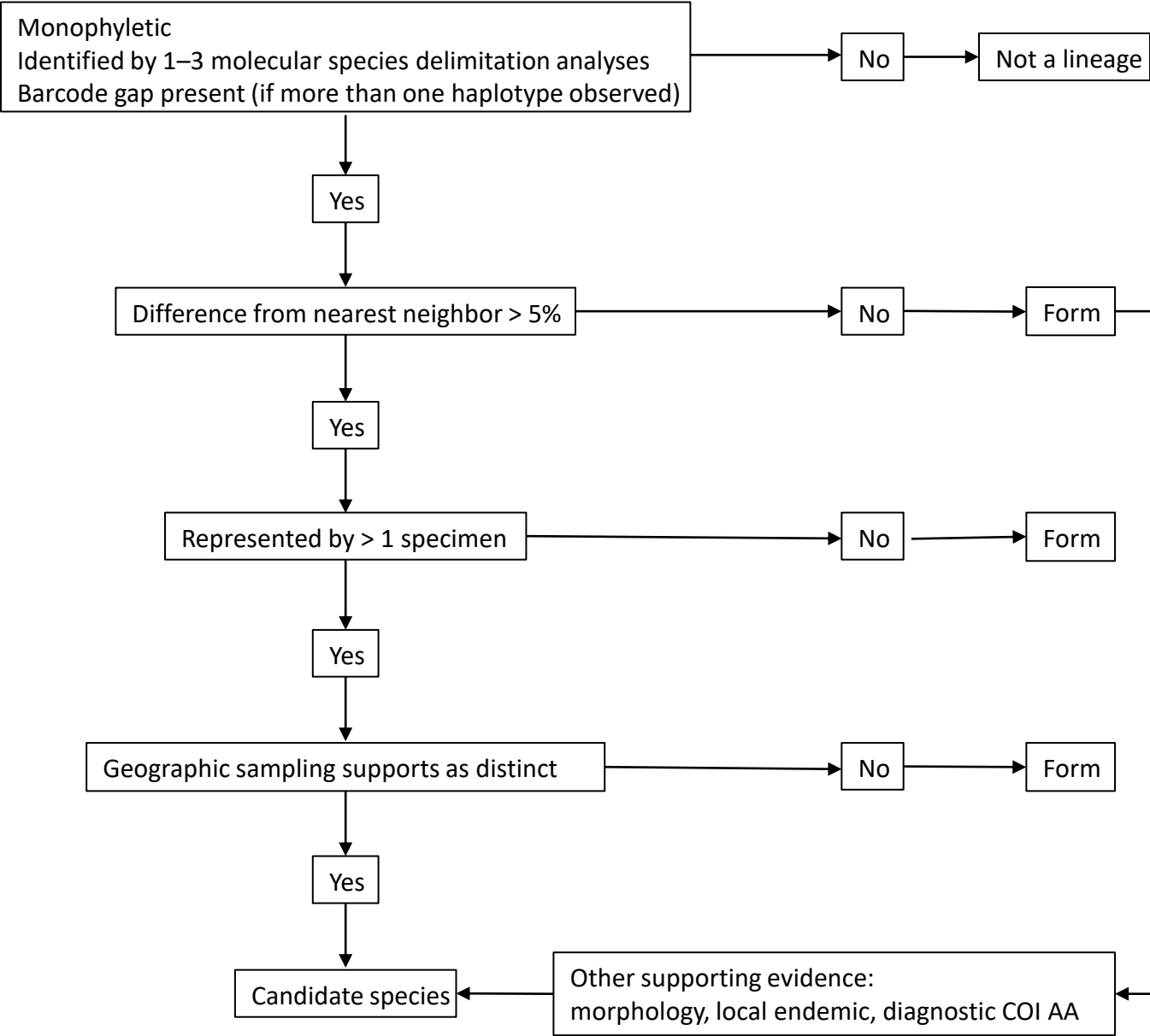

Supplement: Supplementary file 8 — Supplementary Figure S6. [file 41598_2021_1197_MOESM8_ESM.pdf]
