# Supplementary material for: Molecular species delimitation refines the taxonomy of native and nonnative physinine snails in North America
Source: Sci Rep. 2021 Nov 5;11:21739. doi: 10.1038/s41598-021-01197-3 (PMC8571305; doi:10.1038/s41598-021-01197-3)
Supplement: Supplementary file 5 — Supplementary Figure S3. [file 41598_2021_1197_MOESM5_ESM.pdf]

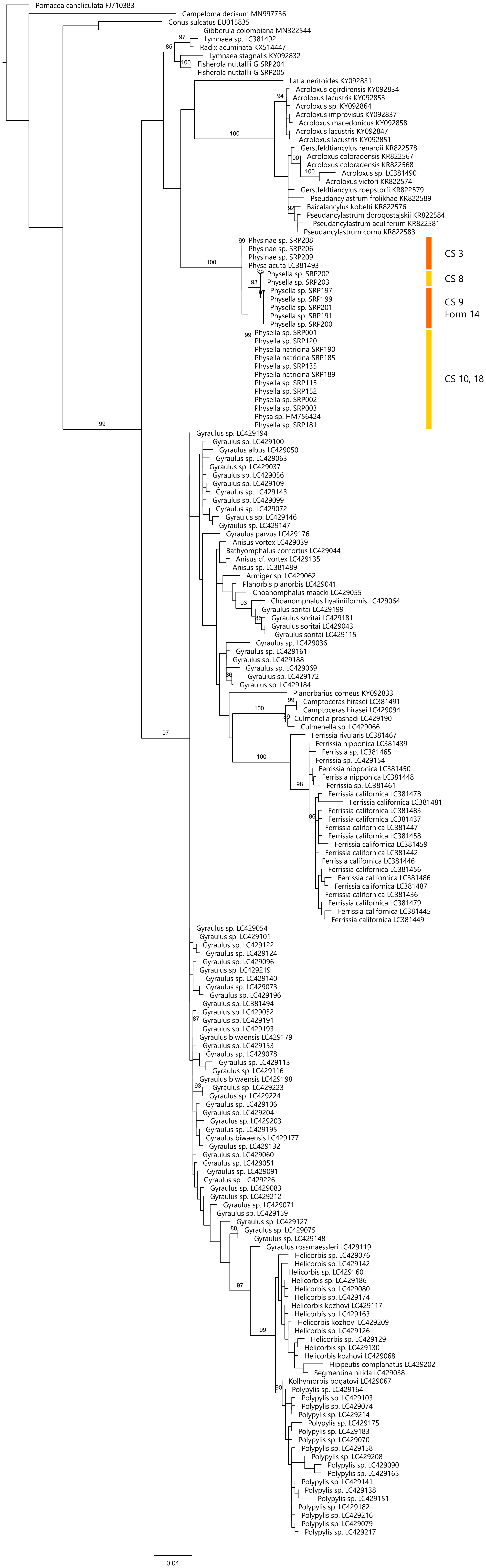

Pomacea canaliculata FJ710383
Campeloma decisum MN997736
Conus sulcatus EU015835
Gibberula colombiana MN322544
Lymnaea sp. LC381492
Radix acuminata KX514447
Lymnaea stagnalis KY092832
Fisherola nuttallii G SRP204
Fisherola nuttallii G SRP205
Latia neritoides KY092831
Acroloxus egirdirensis KY092834
Acroloxus lacustris KY092853
Acroloxus sp. KY092864
Acroloxus improvisus KY092837
Acroloxus macedonicus KY092858
Acroloxus lacustris KY092847
Acroloxus lacustris KY092851
Gerstfeldtiancylus renardii KR822578
Acroloxus coloradensis KR822567
Acroloxus coloradensis KR822568
Acroloxus sp. LC381490
Acroloxus victori KR822574
Gerstfeldtiancylus roepstorfi KR822579
Pseudancylastrum frolikhae KR822589
Baicalancylus kobelti KR822576
Pseudancylastrum dorogostajskii KR822584
Pseudancylastrum aculiferum KR822581
Pseudancylastrum cornu KR822583
Physinae sp. SRP208
Physinae sp. SRP206
Physinae sp. SRP209
Physa acuta LC381493
Physella sp. SRP202
Physella sp. SRP203
Physella sp. SRP197
Physella sp. SRP199
Physella sp. SRP201
Physella sp. SRP191
Physella sp. SRP200
Physella sp. SRP001
Physella sp. SRP120
Physella natricina SRP190
Physella natricina SRP185
Physella sp. SRP135
Physella natricina SRP189
Physella sp. SRP115
Physella sp. SRP152
Physella sp. SRP002
Physella sp. SRP003
Physa sp. HM756424
Physella sp. SRP181
CS 3
CS 8
CS 9
Form 14
CS 10, 18
Gyraulus sp. LC429194
Gyraulus sp. LC429100
Gyraulus albus LC429050
Gyraulus sp. LC429063
Gyraulus sp. LC429037
Gyraulus sp. LC429056
Gyraulus sp. LC429109
Gyraulus sp. LC429143
Gyraulus sp. LC429099
Gyraulus sp. LC429072
Gyraulus sp. LC429146
Gyraulus sp. LC429147
Gyraulus parvus LC429176
Anisus vortex LC429039
Bathyomphalus contortus LC429044
Anisus cf. vortex LC429135
Anisus sp. LC381489
Armiger sp. LC429062
Planorbis planorbis LC429041
Choanomphalus maacki LC429055
Choanomphalus hyaliniiformis LC429064
Gyraulus soritai LC429199
Gyraulus soritai LC429181
Gyraulus soritai LC429043
Gyraulus soritai LC429115
Gyraulus sp. LC429036
Gyraulus sp. LC429161
Gyraulus sp. LC429188
Gyraulus sp. LC429069
Gyraulus sp. LC429172
Gyraulus sp. LC429184
Planorbarius corneus KY092833
Camptoceras hirasei LC381491
Camptoceras hirasei LC429094
Culmenella prashadi LC429190
Culmenella sp. LC429066
Ferrissia rivularis LC381467
Ferrissia nipponica LC381439
Ferrissia sp. LC381465
Ferrissia sp. LC429154
Ferrissia nipponica LC381450
Ferrissia nipponica LC381448
Ferrissia sp. LC381461
Ferrissia californica LC381478
Ferrissia californica LC381481
Ferrissia californica LC381483
Ferrissia californica LC381437
Ferrissia californica LC381447
Ferrissia californica LC381458
Ferrissia californica LC381459
Ferrissia californica LC381442
Ferrissia californica LC381446
Ferrissia californica LC381456
Ferrissia californica LC381486
Ferrissia californica LC381487
Ferrissia californica LC381436
Ferrissia californica LC381479
Ferrissia californica LC381445
Ferrissia californica LC381449
Gyraulus sp. LC429054
Gyraulus sp. LC429101
Gyraulus sp. LC429122
Gyraulus sp. LC429124
Gyraulus sp. LC429096
Gyraulus sp. LC429219
Gyraulus sp. LC429140
Gyraulus sp. LC429073
Gyraulus sp. LC429196
Gyraulus sp. LC381494
Gyraulus sp. LC429052
Gyraulus sp. LC429191
Gyraulus sp. LC429193
Gyraulus biwaensis LC429179
Gyraulus sp. LC429153
Gyraulus sp. LC429078
Gyraulus sp. LC429113
Gyraulus sp. LC429116
Gyraulus biwaensis LC429198
Gyraulus sp. LC429223
Gyraulus sp. LC429224
Gyraulus sp. LC429106
Gyraulus sp. LC429204
Gyraulus sp. LC429203
Gyraulus sp. LC429195
Gyraulus biwaensis LC429177
Gyraulus sp. LC429132
Gyraulus sp. LC429060
Gyraulus sp. LC429051
Gyraulus sp. LC429091
Gyraulus sp. LC429226
Gyraulus sp. LC429083
Gyraulus sp. LC429212
Gyraulus sp. LC429071
Gyraulus sp. LC429159
Gyraulus sp. LC429127
Gyraulus sp. LC429075
Gyraulus sp. LC429148
Gyraulus rossmaessleri LC429119
Helicorbis sp. LC429076
Helicorbis sp. LC429142
Helicorbis sp. LC429160
Helicorbis sp. LC429186
Helicorbis sp. LC429080
Helicorbis sp. LC429174
Helicorbis kozhovi LC429117
Helicorbis sp. LC429163
Helicorbis kozhovi LC429209
Helicorbis sp. LC429126
Helicorbis sp. LC429129
Helicorbis sp. LC429130
Helicorbis kozhovi LC429068
Hippeutis complanatus LC429202
Segmentina nitida LC429038
Kolhymorbis bogatovi LC429067
Polypylis sp. LC429164
Polypylis sp. LC429103
Polypylis sp. LC429074
Polypylis sp. LC429214
Polypylis sp. LC429175
Polypylis sp. LC429183
Polypylis sp. LC429070
Polypylis sp. LC429158
Polypylis sp. LC429208
Polypylis sp. LC429090
Polypylis sp. LC429165
Polypylis sp. LC429141
Polypylis sp. LC429138
Polypylis sp. LC429151
Polypylis sp. LC429182
Polypylis sp. LC429216
Polypylis sp. LC429079
Polypylis sp. LC429217
0.04
