# Supplementary material for: Molecular species delimitation refines the taxonomy of native and nonnative physinine snails in North America
Source: Sci Rep. 2021 Nov 5;11:21739. doi: 10.1038/s41598-021-01197-3 (PMC8571305; doi:10.1038/s41598-021-01197-3)
Supplement: Supplementary file 9 — Supplementary Table S1. [file 41598_2021_1197_MOESM9_ESM.docx]

Supplemental Table 1. Classification of representative members of Physinae, including all taxa reported to be found in Idaho (denoted by an asterisk; primarily from Frest & Johannes 2000) or possibly present in online sequence repositories. Original name indicates the name provided in the species description. The currently valid names are from the Integrated Taxonomic Information System (https://www.itis.gov/), a quasi-governmental U.S. partnership charged with maintaining a database of current and accepted taxonomic names (with apparent misspellings underlined), and MolluscaBase (http://molluscabase.org/index.php), an international collaborative offering an authoritative, current account of all molluscan species. Both databases accessed 14 May 2021.

| Original name | Integrated Taxonomic Information System | MolluscaBase | Type location |
| --- | --- | --- | --- |
| *Aplexa microstriata* Chamberlin & Berry 1930 | *Physella microstriata* | *Utahphysa microstriata* | Fish Lake, UT |
| *Bulla fontinalis*  Linnaeus 1758 | *Physa fontinalis* | *Physa fontinalis* | Unavailable |
| *Physa acuta**  Draparnaud 1805 | *Physella acuta* | *Physella acuta* | Garonne River, France |
| *Physa ampullacea**  Gould in Binney 1885 | **Not recognized** | *Physella gyrina* | Oregon |
| *Physa anatina*  Lea 1864 | **Not recognized** | *Physella acuta* | Arkansas River, KS |
| *Physa ancillaria*  Say 1825 | *Physella ancillaria* | *Physella ancillaria* | Delaware River, PA |
| *Physa ancillaria magnalacustris* Walker 1901 | *Physella magnalacustris* | **Taxon inquirendum^1^** | Lake Michigan, Frankfort, MI |
| *Physa aurea*  Lea 1838 | *Physella gyrina aurea* | *Physella gyrina* | Hot Springs, Bath County, VA |
| *Physa bermudezi*  Aguayo 1935 | *Physella bermudezi* | **Taxon inquirendum** | Ciénaga de Zapata, Cuba |
| *Physa billingsii*  Heron 1880 | **Not recognized** | *Physella acuta* | Castor River, ON |
| *Physa bottimeri*  Clench 1924 | *Physella bottimeri* | **Taxon inquirendum** | Comanche Springs, TX |
| *Physa boucardi*  Crosse & Fischer 1881 | *Physella boucardi* | **Taxon inquirendum** | Lake Texcoco, Mexico |
| *Physa brevispira*  Lea 1864 | **Not recognized** | *Physella acuta* | Ottawa River, ON |
| *Physa carolinae*  Wethington, Wise & Dillon 2009 | *Physa carolinae* | *Physella carolinae* | Huger Creek spring, SC |
| *Physa columbiana**  Hemphill 1890 | *Physella columbiana* | *Physella columbiana* | Columbia River, Astoria, OR |
| *Physa concolor*  Haldeman 1841 | *Physella gyrina* | *Physella gyrina* | Oregon |
| *Physa conoidea*  Fischer & Crosse 1886 | *Physella conoidea* | *Physella acuta* | Lake Texcoco, Mexico |
| *Physa cooperi**  Tryon 1865 | *Physella cooperi* | **Taxon inquirendum** | Cowhead Lake, CA |
| *Physa costata*  Newcomb 1861 | *Physella costata* | *Physella costata* | Clear Lake, CA |
| *Physa cubensis**  Pfeiffer 1839 | *Physella cubensis* | *Physella acuta* | Cuba |
| *Physa elongata**  Say 1821 | *Aplexa elongata* | *Sibirenauta elongata* | Mississippi River, St. Louis, MO |
| *Physa globosa*  Haldeman 1842 | *Physella globosa* | *Physella globosa* | Nolichucky River, TN |
| *Physa gyrina**  Say 1821 | *Physella gyrina* | *Physella gyrina* | Boyer River, IA |
| *Physa heterostropha*  Say 1817 | *Physella heterostropha* | *Physella acuta* | Delaware River, PA |
| *Physa hordacea*  Lea 1864 | *Physella hordacea* | *Physella hordacea* | Vancouver Island, WA |
| *Physa humerosa*  Gould 1855 | *Physella humerosa* | **Taxon inquirendum** | Colorado Desert, CA |
| *Physa integra*  Haldeman 1841 | *Physella integra* | *Physella acuta* | Indiana |
| *Physa jennessi*  Dall 1919 | *Physa jennessi* | *Beringophysa jennessi* | Ponds, Bernard Harbour, NWT |
| *Physa jennessi athearni*  Clarke 1973 | *Physa jennessi* | *Physella gyrina* | Horseshoe Lake, AB |
| *Physa jennessi skinneri*  Clarke 1973 | *Physa skinneri* | **Not recognized** | Beaver County, OK |
| *Physa johnsoni*  Clench 1926 | *Physella johnsoni* | *Physella johnsoni* | Middle Spring, Banff, AB |
| *Physa lordi utahensis*  Clench 1925 | *Physella utahensis* | *Physella gyrina* | Utah Lake, UT |
| *Physa lordi**  Baird 1863 | *Physella lordi* | *Physella lordi* | Lake Osoyoos, BC |
| *Physa megalochlamys**  Taylor 1988 | *Physa megalochlamys* | *Physa megalochlamys* | Teton County, WY |
| *Physa mexicana*  Philippi in Küster 1841 | *Physella mexicana* | *Physella acuta* | Mexico |
| *Physa microstoma*  Haldeman 1840 | **Not recognized** | *Physella microstoma* | Unavailable |
| *Physa natricina**  Taylor 1988 | *Physa natricina* | *Physella natricina* | Snake River, Gooding County, ID |
| *Physa niagarensis*  *Lea 1864* | **Not recognized** | *Physella acuta* | Niagara River, NY |
| *Physa nuttallii**  Lea 1864 | *Physella propinqua* | *Physella gyrina* | Columbia River, OR |
| *Physa osculans*  Haldeman 1841 | *Physella osculans* | *Physella osculans* | Mexico |
| *Physa parkeri*  Currier in DeCamp 1881 | *Physella parkeri* | *Physella parkeri* | Houghton Lake, MI |
| *Physa pomilia*  Conrad 1834 | *Physella pomilia* | *Physella pomilia* | Randons Creek, AB |
| *Physa pomilia hendersoni* Clench 1925 | *Physella hendersoni* | *Physella hendersoni* | Combahee River, SC |
| *Physa propinqua**  Tryon 1865 | *Physella propinqua* | **Taxon inquirendum** | Jordan River, ID |
| *Physa sayii*  Tappan 1839 | *Physella heterostropha* | *Physella gyrina* | Unavailable |
| *Physa sibirica*  Westerlund 1876 | *Physa siberica* | *Sibirenauta sibirica* | Siberia, Russia |
| *Physa skinneri**  Taylor 1954 | *Physa skinneri* | *Physa skinneri* | Beaver County, OK |
| *Physa spelunca*  Turner & Clench 1974 | *Physella spelunca* | *Physella spelunca* | Lower Kane Cave, WY |
| *Physa squalida*  Morelet 1851 | *Physella squalida* | **Taxon inquirendum** | Marshes, Tabasco, Mexico |
| *Physa traskii*  Lea 1864 | *Physella traski* | **Taxon inquirendum** | Los Angeles River, CA |
| *Physa triticea**  Lea 1856 | **Not recognized** | *Physella gyrina* | Shasta County, CA |
| *Physa venusta**  Lea 1864 | **Not recognized** | *Physella gyrina* | Fort Vancouver, WA |
| *Physa vernalis*  Taylor & Jokinen 1984 | *Physa vernalis* | *Physa vernalis* | Windham Township, CT |
| *Physa vinosa*  Gould 1847 | *Physella vinosa* | *Physella vinosa* | Lake Superior, MI |
| *Physa virgata*  Gould 1855 | *Physella virgata* | *Physella acuta* | Gila River, AZ |
| *Physa virginea**  Gould 1847 | *Physella virginea* | **Taxon inquirendum** | Mountain Lake, CA |
| *Physa wolfiana*  Lea 1869 | **Not recognized** | *Physella gyrina* | Hot Sulphur Springs, CO |
| *Physa zionis*  Pilsbry 1926 | *Physella zionis* | *Physella zionis* | NF Virgin River basin, UT |
| *Physella latchfordi*  Baker 1928 | *Physella gyrina* | *Physella latchfordi* | Meechs Lake, QC |
| *Physella winnipegensis*  Pip 2004 | *Physella winnipegensis* | *Physella acuta* | Lake Winnepeg, MB |
| *Physella wrighti*  Te & Clarke 1985 | *Physella wrighti* | *Physella gyrina* | Liard Hot Springs, BC |

^1^Taxon inquirendum refers to an incompletely defined taxon for which the taxonomic validity is uncertain or disputed by different experts, or a taxon that is impossible to identify.
