# Supplementary material for: Molecular species delimitation refines the taxonomy of native and nonnative physinine snails in North America
Source: Sci Rep. 2021 Nov 5;11:21739. doi: 10.1038/s41598-021-01197-3 (PMC8571305; doi:10.1038/s41598-021-01197-3)
Supplement: Supplementary file 6 — Supplementary Figure S4. [file 41598_2021_1197_MOESM6_ESM.pdf]

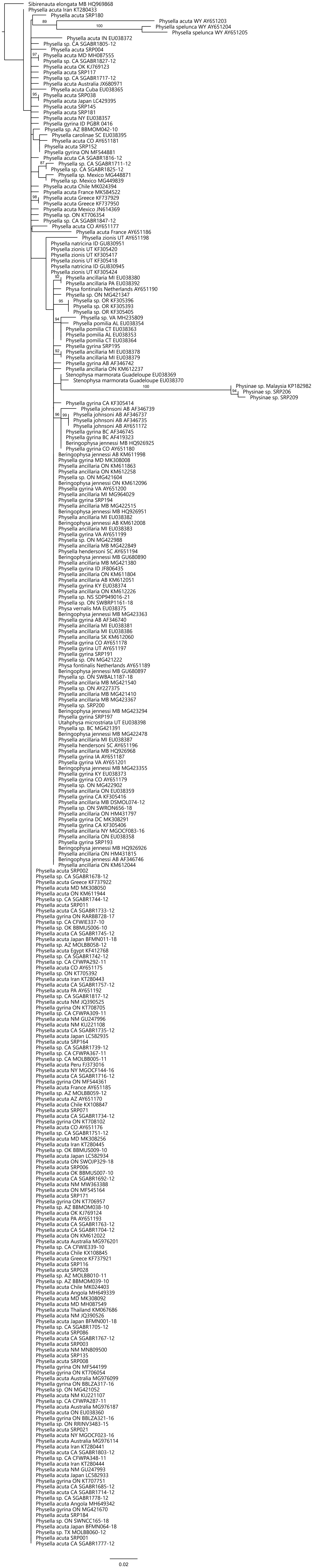
Sibirenauta elongata MB HQ969868
Physella acuta Iran KT280433
Physella acuta SRP180
89
Physella acuta WY AY651203
100
Physella spelunca WY AY651204
Physella spelunca WY AY651205
Physella acuta IN EU038372
Physella sp. CA SGABR1805-12
Physella acuta SRP004
97
Physella acuta MD MH087555
Physella sp. CA SGABR1827-12
Physella acuta OK KJ769123
Physella acuta SRP117
Physella sp. CA SGABR1717-12
Physella acuta Australia JX680971
Physella acuta Cuba EU038365
95
Physella acuta SRP038
Physella acuta Japan LC429395
Physella acuta SRP145
Physella acuta SRP181
Physella acuta NY EU038357
Physella gyrina ID PGBR 0416
Physella sp. AZ BBMOM042-10
Physella carolinae SC EU038395
Physella acuta CO AY651181
Physella acuta SRP152
Physella gyrina ON MF544881
Physella acuta CA SGABR1816-12
87
Physella sp. CA SGABR1711-12
Physella sp. CA SGABR1825-12
Physella sp. Mexico MG448871
Physella sp. Mexico MG449839
Physella acuta Chile MK024394
Physella acuta France MK584522
98
Physella acuta Greece KF737929
Physella acuta Greece KF737950
Physella acuta Mexico JN614369
Physella sp. ON KT706354
Physella sp. CA SGABR1847-12
Physella acuta CO AY651177
Physella acuta France AY651186
Physella zionis UT AY651198
Physella natricina ID GU830951
Physella zionis UT KF305420
Physella zionis UT KF305417
Physella zionis UT KF305418
Physella natricina ID GU830945
Physella zionis UT KF305424
92
Physella ancillaria MI EU038380
Physella ancillaria PA EU038392
Physa fontinalis Netherlands AY651190
Physella sp. ON MG421347
95
Physella sp. OR KF305396
Physella sp. OR KF305393
Physella sp. OR KF305405
94
Physella sp. VA MH235809
Physella pomilia AL EU038354
Physella pomilia CT EU038363
Physella pomilia AL EU038353
Physella pomilia CT EU038364
Physella gyrina SRP195
92
Physella ancillaria MI EU038378
Physella ancillaria MI EU038379
Physella gyrina AB AF346742
Physella ancillaria ON KM612237
Stenophysa marmorata Guadeloupe EU038369
Stenophysa marmorata Guadeloupe EU038370
100
Physinae sp. Malaysia KP182982
94
Physinae sp. SRP206
Physinae sp. SRP209
Physella gyrina CA KF305414
Physella johnsoni AB AF346739
96
99
Physella johnsoni AB AF346737
Physella johnsoni AB AF346735
Physella johnsoni AB AY651172
Physella gyrina BC AF346745
Physella gyrina BC AF419323
Beringophysa jennessi MB HQ926925
Physella gyrina CO AY651180
Beringophysa jennessi AB KM611998
Physella gyrina MD MK308008
Physella ancillaria ON KM611863
Physella ancillaria ON KM612258
Physella sp. ON MG421604
Beringophysa jennessi ON KM612096
Physella gyrina VA AY651200
Physella ancillaria MI MG964029
Physella gyrina SRP194
Physella ancillaria MB MG422515
Beringophysa jennessi MB HQ926951
Physella ancillaria MI EU038382
Beringophysa jennessi AB KM612008
Physella ancillaria MI EU038383
Physella gyrina VA AY651199
Physella sp. ON MG422988
Physella ancillaria MB MG422849
Physella hendersoni SC AY651194
Beringophysa jennessi MB GU680890
Physella ancillaria MB MG421380
Physella gyrina ID JF806435
Physella ancillaria ON KM611804
Physella ancillaria AB KM612051
Physella gyrina KY EU038374
Physella ancillaria ON KM612226
Physella sp. NS SDP949016-21
Physella sp. ON SWBRP1161-18
Physa vernalis MA EU038375
Beringophysa jennessi MB MG423363
Physella gyrina AB AF346740
Physella ancillaria MI EU038381
Physella ancillaria MI EU038386
Physella ancillaria SK KM612060
Physella gyrina CO AY651178
Physella gyrina UT AY651197
Physella gyrina SRP191
Physella sp. ON MG421222
Physa fontinalis Netherlands AY651189
Beringophysa jennessi MB GU680897
Physella sp. ON SWBAL1187-18
Physella ancillaria MB MG421540
Physella sp. ON AY227375
Physella ancillaria MB MG421410
Physella ancillaria MB MG423367
Physella sp. SRP200
Beringophysa jennessi MB MG423294
Physella gyrina SRP197
Utahphysa microstriata UT EU038398
Physella sp. BC MG421391
Beringophysa jennessi MB MG422478
Physella ancillaria MI EU038387
Physella hendersoni SC AY651196
Physella ancillaria MB HQ926968
Physella gyrina IA AY651187
Physella gyrina VA AY651201
Beringophysa jennessi MB MG423355
Physella gyrina KY EU038373
Physella gyrina CO AY651179
Physella sp. ON MG422902
Physella ancillaria ON EU038359
Physella gyrina CA KF305416
Physella ancillaria MB DSMOL074-12
Physella sp. ON SWRON656-18
Physella ancillaria ON HM431797
Physella gyrina DC MK308291
Physella gyrina CA KF305406
Physella ancillaria NY MGOCF083-16
Physella ancillaria ON EU038358
Physella gyrina SRP193
Beringophysa jennessi MB HQ926926
Physella ancillaria ON HM431815
Beringophysa jennessi AB AF346746
Physella ancillaria ON KM612044
Physella acuta SRP002
Physella sp. CA SGABR1678-12
Physella acuta Greece KF737922
Physella acuta MD MK308050
Physella acuta ON KM611944
Physella sp. CA SGABR1744-12
Physella acuta SRP011
Physella acuta CA SGABR1733-12
Physella gyrina ON RARBB728-17
Physella sp. CA CFWIE337-10
Physella sp. OK BBMUS006-10
Physella acuta CA SGABR1745-12
Physella acuta Japan BFMN011-18
Physella sp. AZ MOLBB058-12
Physella acuta Egypt KF412768
Physella sp. CA SGABR1742-12
Physella sp. CA CFWPA292-11
Physella acuta CO AY651175
Physella sp. ON KT705392
Physella acuta Iran KT280443
Physella acuta CA SGABR1757-12
Physella acuta PA AY651192
Physella sp. CA SGABR1817-12
Physella acuta NM JQ390525
Physella gyrina ON KT708705
Physella sp. CA CFWPA309-11
Physella acuta NM GU247996
Physella acuta NM KU221108
Physella acuta CA SGABR1735-12
Physella acuta Japan LC582935
Physella acuta SRP164
Physella sp. CA SGABR1739-12
Physella sp. CA CFWPA367-11
Physella sp. CA MOLBB005-11
Physella acuta Peru FJ373016
Physella acuta NY MGOCF144-16
Physella acuta CA SGABR1716-12
Physella gyrina ON MF544361
Physella acuta France AY651185
Physella sp. AZ MOLBB059-12
Physella acuta AZ AY651170
Physella acuta Chile KX108847
Physella acuta SRP071
Physella acuta CA SGABR1734-12
Physella gyrina ON KT708102
Physella acuta CO AY651176
Physella sp. CA SGABR1751-12
Physella acuta MD MK308256
Physella acuta Iran KT280445
Physella sp. OK BBMUS009-10
Physella acuta Japan LC582934
Physella acuta ON SWOJP329-18
Physella acuta SRP006
Physella acuta OK BBMUS007-10
Physella acuta CA SGABR1692-12
Physella acuta NM MW363388
Physella acuta ON MF545164
Physella acuta SRP171
Physella gyrina ON KT706957
Physella sp. AZ BBMOM038-10
Physella acuta OK KJ769124
Physella acuta PA AY651193
Physella acuta CA SGABR1763-12
Physella acuta CA SGABR1704-12
Physella acuta ON KM612022
Physella acuta Australia MG976201
Physella sp. CA CFWIE339-10
Physella acuta Chile KX108845
Physella acuta Greece KF737921
Physella acuta SRP116
Physella acuta SRP028
Physella sp. AZ MOLBB010-11
Physella sp. AZ BBMOM039-10
Physella acuta Chile MK024403
Physella acuta Angola MH649339
Physella acuta MD MK308092
Physella acuta MD MH087549
Physella acuta Thailand KM067686
Physella acuta NM JQ390526
Physella acuta Japan BFMN001-18
Physella sp. CA SGABR1705-12
Physella acuta SRP086
Physella acuta CA SGABR1767-12
Physella acuta SRP003
Physella acuta NM MN809500
Physella acuta SRP135
Physella acuta SRP008
Physella gyrina ON MF544199
Physella gyrina ON KT706054
Physella acuta Australia MG976099
Physella gyrina ON BBLZA317-16
Physella sp. ON MG421052
Physella acuta NM KU221107
Physella sp. CA CFWPA287-11
Physella acuta Australia MG976187
Physella acuta ON EU038360
Physella gyrina ON BBLZA321-16
Physella sp. ON RRINV3483-15
Physella acuta SRP021
Physella acuta NY MGOCF023-16
Physella acuta Australia MG976114
Physella acuta Iran KT280441
Physella acuta CA SGABR1803-12
Physella sp. CA CFWPA348-11
Physella acuta Iran KT280444
Physella acuta NM GU247993
Physella acuta Japan LC582933
Physella gyrina ON KT707751
Physella acuta CA SGABR1685-12
Physella acuta CA SGABR1714-12
Physella sp. CA SGABR1778-12
Physella acuta Angola MH649342
Physella gyrina ON MG421670
Physella acuta SRP184
Physella sp. ON SWNCC165-18
Physella acuta Japan BFMN064-18
Physella sp. TX MOLBB060-12
Physella acuta SRP001
Physella acuta CA SGABR1777-12
0.02
