# Supplementary material for: Molecular species delimitation refines the taxonomy of native and nonnative physinine snails in North America
Source: Sci Rep. 2021 Nov 5;11:21739. doi: 10.1038/s41598-021-01197-3 (PMC8571305; doi:10.1038/s41598-021-01197-3)

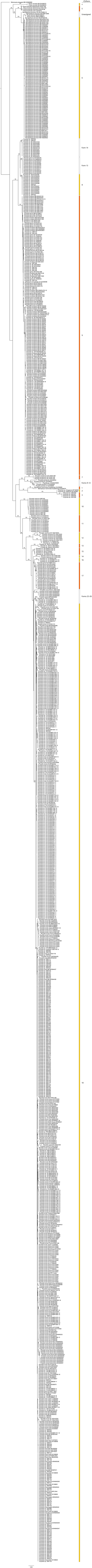

Supplement: Supplementary file 7 — Supplementary Figure S5. [file 41598_2021_1197_MOESM7_ESM.pdf]
